# Executive Function, Theory of Mind, and Conduct-Problem Symptoms in Middle Childhood

**DOI:** 10.3389/fpsyg.2020.00539

**Published:** 2020-04-02

**Authors:** Gina Austin, Rebecca Bondü, Birgit Elsner

**Affiliations:** ^1^Department of Psychology, University of Potsdam, Potsdam, Germany; ^2^Department of Psychology, Psychologische Hochschule Berlin, Berlin, Germany

**Keywords:** executive functions, theory of mind, conduct-problem symptoms, middle childhood, longitudinal

## Abstract

Studies show relations between executive function (EF), Theory of Mind (ToM), and conduct-problem (CP) symptoms. However, many studies have involved cross-sectional data, small clinical samples, pre-school children, and/or did not consider potential mediation effects. The present study examined the longitudinal relations between EF, ToM abilities, and CP symptoms in a population-based sample of 1,657 children between 6 and 11 years (T1: *M* = 8.3 years, T2: *M* = 9.1 years; 51.9% girls). We assessed EF skills and ToM abilities via computerized tasks at first measurement (T1), CP symptoms were rated via parent questionnaires at T1 and approximately 1 year later (T2). Structural-equation models showed a negative relation between T1 EF and T2 CP symptoms even when controlling for attention-deficit hyperactivity disorder (ADHD) symptoms and other variables. This relation was fully mediated by T1 ToM abilities. The study shows how children’s abilities to control their thoughts and behaviors and to understand others’ mental states interact in the development of CP symptoms.

## Introduction

Child conduct problems (CP) refer to an array of oppositional, aggressive, or deviant behavior that manifests recurrently over long periods of time. These problems are characterized by the violation of others’ rights, age-appropriate social expectations, and/or (in)formal social norms (Lahey and Waldman, 2007). The long-term expectations of social adjustment by individuals showing CP are poor, particularly when the onset occurs early in life (Moffitt, 1993): They are more likely to quit school, engage in juvenile delinquency, and develop antisocial personality disorder as adults (Moffitt, 2006, for reviews). Hence, CP place a burden on those affected and their social environment and cause enormous costs for public health services. Therefore, it is essential to gain a more profound understanding of the early risk factors and developmental mechanisms that lead to childhood CP symptoms.

Neurocognitive deficits, including impairments in executive function (EF), are important in the development and persistence of CP (Moffitt, 1993, 2006; Ogilvie et al., 2011, for a meta-analysis). EF refers to a set of self-regulatory cognitive processes that allow the individual to control thoughts and behaviors. The exact definitions and the assumed number of these processes as well as their combinations differ (Morra et al., 2018, for an overview), but mostly EF are thought to encompass three interrelated but dissociable subcomponents: cognitive flexibility (or: attention shifting), inhibition, and (updating of) working memory (Miyake et al., 2000; Garon et al., 2008; Karr et al., 2018; Morra et al., 2018). EF skills are pivotal for socially appropriate behavior (Morgan and Lilienfeld, 2000; Ogilvie et al., 2011) and deficits in these skills were associated with an early onset and a high persistence of CP (Moffitt, 1993).

A correlate of EF and CP symptoms is Theory of Mind (ToM), the ability to attribute and infer others’ mental states (Frith and Frith, 2007). EF skills and ToM abilities are consistently positively related, particularly in preschool years (Devine and Hughes, 2014, for a review; Hughes, 1998; Lagattuta et al., 2016; Wade et al., 2018, for a review). The ability to represent others’ mental states is a powerful tool to predict and explain their behavior (Moore and Frye, 1991) and seems a prerequisite for appropriate social functioning (Astington, 2003; Hughes and Leekam, 2004, for reviews). Accordingly, some studies found lower ToM abilities in children who showed antisocial or aggressive behavior (Capage and Watson, 2001; Austin et al., 2017).

Based on such findings, it was argued that the relation of EF skills and CP symptoms may be mediated by ToM abilities (Riggs et al., 2006; Razza and Blair, 2009). For example, EF skills predicted ToM abilities in a clinical group of preschoolers with conduct disorder, suggesting direct *and* indirect associations between EF and CP (Hughes et al., 1998). So far, this model was only tested in preschool children (Razza and Blair, 2009), but EF (Huizinga et al., 2006) and ToM (Devine and Hughes, 2012) continue to develop during middle childhood and externalizing problem behavior peaks at school entry (Mesman et al., 2001). At the same time, transition to school is characterized by increasing social interactions and higher expectations to perform. Hence, particularly this developmental stage requires advanced self-regulatory and social skills and poses challenges to children’s existing EF and ToM abilities (Lalonde and Chandler, 1995). Thus, it seems important to examine the influence of EF skills and ToM abilities on the emergence of CP symptoms in middle childhood. Furthermore, samples from the general population should be examined in order to test whether the relations between EF, ToM, and CP in clinical samples can also be found in non-selected samples with low CP symptoms and whether cross-sectional findings are replicated with longitudinal data.

The present study, therefore, examined the longitudinal links of EF and ToM with CP symptoms in a large sample of elementary school-aged children from the general population. We also tested the assumption that ToM mediates the relation between EF and CP symptoms in order to add to a better understanding of the processes that may promote CP symptoms in middle childhood.

### CP Symptoms and Their Links With EF and ToM

Prevalence rates for conduct disorder vary between 7% to over 10% (Ravens-Sieberer et al., 2008). Affected children account for large proportions of norm violations in middle childhood (Moffitt, 1993). CP symptoms, however, are heterogeneous; they cover a wide range of deviant behavior, including lying, stealing, eloping, disobedience, cruelty to animals, or aggression; and manifest differently in various age groups (Lahey et al., 2000). Thus, prevalence rates for single expressions of CP symptoms are much higher, even among children from the general public, without necessarily qualifying for a diagnosis of conduct disorder. Many children outgrow these maladaptive behavior patterns. But stability rates are high, a large percentage of individuals with CP in childhood shows antisocial personality disorder as adults, and sometimes CP symptoms only develop in adolescence (Moffitt, 2006).

The development of EF, which is associated with CP (Moffitt, 1993), advances rapidly during the first years of life, and continues during middle childhood (Huizinga et al., 2006) into adolescence (Luciana et al., 2005). EF is fundamental for socially appropriate behavior. It comprises the ability to flexibly switch attention between and flexibly respond to different social stimuli, to inhibit impulsive behavior, as well as to code, monitor, and update social information so that the individual can follow fast-paced social situations (Bierman et al., 2008).

In adults, there is considerable evidence for negative relations between EF skills and CP or related domains, such as antisocial behavior (Ogilvie et al., 2011, for a meta-analysis). Studies with children suggest similar relations: A meta-analysis in preschoolers showed that children with externalizing problem behavior displayed lower EF skills (Schoemaker et al., 2013). The few longitudinal studies on the subject in non-clinical samples confirmed the negative relation between EF skills and CP in childhood (Riggs et al., 2003; Sulik et al., 2015; Holmes et al., 2016). Many previous studies, however, were cross-sectional and conducted in clinical samples (Fahie and Symons, 2003). Thus, it seems important to examine the link between EF skills and CP symptoms in community samples in order to gain a deeper insight into the mechanisms of less extreme behavior problems so that appropriate interventions for the general population can be developed.

It has also been argued, however, that these findings need to be treated with caution due to high comorbidity rates between attention-deficit hyperactivity disorder (ADHD) and conduct disorder (Hinshaw et al., 1993). Because individuals diagnosed with ADHD have consistently been found to show low EF (Oosterlaan et al., 2005; Rubia, 2011, for a review), particularly the inhibition of impulsive actions (Fahie and Symons, 2003; Toupin et al., 2000), ADHD symptoms may confound the relation between EF skills and CP symptoms. Indeed, in some studies, the relation between EF and CP was small once ADHD was controlled for (Pennington and Ozonoff, 1996; Oosterlaan et al., 2005; Brocki et al., 2007; for a meta-analysis; Thorell and Wåhlstedt, 2006). Others found relations between EF skills and CP even after controlling for ADHD (symptoms) in preschool age (Raaijmakers et al., 2008), middle childhood (Toupin et al., 2000), adolescence (Hobson et al., 2011; Séguin et al., 1999), and adults (Ogilvie et al., 2011, for a meta-analysis). Thus, it is important to consider the potentially confounding role of ADHD symptoms in the relation between EF skills and CP symptoms. The present study additionally examines the potential mediating role of ToM.

ToM skills also develop from infancy into adolescence (Lalonde and Chandler, 2002). ToM deficits have been associated with externalizing problem behavior (Hughes et al., 1998; Hughes and Ensor, 2006), callous unemotional behavior (Dadds et al., 2009; Jones et al., 2010), and CP (in 9–11 years olds Sharp, 2008). Studies also revealed positive associations between ToM abilities and prosocial behavior in 2–12 years old (Imuta et al., 2016, for a meta-analysis). Other studies, however, found positive links between ToM and bullying (Sutton et al., 1999; Caravita et al., 2010) or relational aggression (Gomez-Garibello and Talwar, 2015), and no link between ToM abilities and conduct disorder (Happé and Frith, 1996). These controversial findings, however, mainly rely on cross-sectional data and require replication in larger samples and longitudinal studies.

### The Potential Mediating Role of ToM Abilities

Research showed a robust positive relation between EF skills and ToM abilities during the preschool years (Devine and Hughes, 2014; Wade et al., 2018, for reviews), through middle childhood (Austin et al., 2014; Miller, 2009), and into adolescence (Dumontheil et al., 2010). EF is often considered as crucial for the ability to infer and understand the mental states of others. In line with this reasoning, behavioral research in middle childhood provided stronger support for the notion that EF promotes and precedes ToM than for the opposite (Austin et al., 2014; Devine and Hughes, 2014, for a review; Wade et al., 2018, for a review). Cognitive neuroscientific research suggests that the sequence is not as clear cut and that EF skills and ToM abilities are to some extend independent. There is also evidence that ToM abilities are the prerequisite for more complex EF skills, such as planning and behavioral control, which require an understanding and the consideration of others’ mental states (Wade et al., 2018, for a review). We, however, based our predictions on the dominance of findings (including our own previous research, Austin et al., 2014), supporting the view that EF skills should precede ToM abilities, and also assumed that EF skills should precede ToM abilities.

Regarding the potential link of EF skills and ToM abilities in predicting CP symptoms, it has been suggested that EF skills influence social competence (negatively related to CP symptoms) directly and indirectly through ToM (Riggs et al., 2006). That is, sophisticated EF skills may enable and foster the development of ToM abilities, for example because cognitive flexibility and inhibition are required for changing perspective. This, in turn, may add to reducing the likelihood of CP symptoms, because taking others’ perspective may for example enable the individual to adequately adapt own behavior to others’ intentions and needs.

Given the specific demands of school entry, the mediation model may particularly apply to middle childhood, because in this age-range, children face numerous developmental tasks, such as building new friendships or building relationships with teachers, that require both basal EF skills and ToM abilities. However, the only longitudinal study so far was performed in preschool-age (Razza and Blair, 2009), and failed to support the model when the stability of social competence was accounted for. Instead, ToM abilities were a unique predictor of social competence, independent of EF skills. Two cross-sectional studies yielded similar results in 2-year olds (Hughes and Ensor, 2006), but did not find a significant association between ToM and social problems in older children (mean age: 6.5 years) after considering EF (Fahie and Symons, 2003). Hence, the current state of research provides mixed evidence for the simultaneous effects of EF and ToM on behavioral problems during childhood, and no longitudinal study has tested the potential mediating effect of ToM abilities on the link between EF skills and CP symptoms in middle childhood.

### The Present Study

The present study addressed the theoretical and methodological issues outlined above by investigating (1) the influence of EF skills on CP symptoms while controlling for the influence of ADHD symptoms and (2) the mediating role of ToM abilities in the relation between early EF skills and later CP symptoms longitudinally at two points of measurement (about one year apart) in a large population-based sample of children aged 6–11 years. By including the complete period of middle childhood during which strong increases of ToM and EF may be expected, we were able to examine whether results would refer to the entire age range. We used a broad set of computerized and non-computerized tasks to assess ToM and EF subcomponents (flexibility, inhibition, and executive aspects of the working memory). We controlled for ADHD symptoms (Thorell and Wåhlstedt, 2006) and fluid intelligence, because EF skills and CP symptoms are closely related to cognitive abilities (Lynam et al., 1993). We hypothesized that (1) EF skills would negatively predict later CP symptoms over and beyond ADHD symptoms, (2) EF skills would be positively related to ToM abilities, which, in turn, would negatively predict later CP symptoms, and (3) there should be a significant indirect effect of EF skills on CP symptoms via ToM abilities. This way, our study aimed to clarify the interacting roles of behavioral control and of mental understanding for the development of CP symptoms in middle childhood in a population-based sample.

## Materials and Methods

### Participants

At first measurement (T1), the sample consisted of *N* = 1,657 children (52.1% girls) aged 6-11 years (*M* = 8.3 years, *SD* = 0.95); for *N* = 1,339 of these children, parents provided questionnaire data. At second measurement (T2), *N* = 1,619 children (51.9% girls), aged 7–12 years (*M* = 9.1 years, *SD* = 0.92) participated; for *N* = 1,160 of these children, parents provided data. The interval between the first assessment at T1, which took place between February and December 2012 when children attended first to fourth grade (1st grade: 26.7%, 2nd grade: 33.2%, 3rd grade: 31.7%, 4th grade: 8.3%), and the second assessment at T2, which took place between January and December 2013 when children attended first to fifth grade (1st grade: 0.4%, 2nd grade: 29.2%, 3rd grade: 35.0%, 4th grade: 35.2%, 5th grade: 0.2%), was about 9 months on average (*M*delay = 272.8 days, *SD* = 55.1). Participants were recruited for a study on intrapersonal risk factors for maladaptive development and behavioral problems in childhood and adolescence. First, schools were contacted and asked for participation. Out of these, 33 primary schools in the federal state of Brandenburg, Germany, from rural and urban areas as well as from different socio-economic backgrounds agreed to participate. Then parents of children in classes 1–3 were approached and asked for their participation. Of the participating mothers, 21.6% reported university entrance qualification and 33.7% a university degree at the time of the first measurement. Of the participating fathers, 13.5% reported university entrance qualification and 36.7% a university degree.

### Measures

#### Flexibility

In the Attention Shifting Task (Röthlisberger et al., 2010; adapted from Zimmermann et al., 2002), children were simultaneously shown a multi-colored and a single-colored fish on the left-hand and right-hand side of a computer screen. Children were instructed to alternately “feed” the two kinds of fish by pressing the X-key for the left-hand fish or the M-key for the right-hand fish on a QWERTY keyboard. Across 46 trials (inter-stimulus intervals: 300–700 ms), the two kinds of fish appeared randomly at either side, requiring participants to flexibly adapt their pattern of key presses. Switching between stimuli and adapting to changing situations as required in the present task correspondes to the definition of flexibility and flexibly switching attention (Morra et al., 2018). Pressing a button resulted in a feedback sound. We calculated the number of correct responses in the 22 switch trials in which children had to change their response pattern (i.e., from pressing left/right to left/left or right/right).

#### Inhibition

In the Fruit Stroop Task (Röthlisberger et al., 2010; adapted from Archibald and Kerns, 1999), children were presented with 4 paper sheets, each depicting 25 stimuli. Children should name the color of the items (pages 1 and 2) or the color that the items would normally have (pages 3 and 4) as fast as possible (and – if applicable – to correct errors immediately). The depicted stimuli were: on page 1, colored rectangles (red, green, blue, yellow); the other pages depicted the same fruits and vegetables: on page 2 in their typical colors (banana–yellow, lettuce–green, strawberry–red, plum–blue); on page 3 in gray; on page 4 colored incorrectly (e.g., banana–blue). Thus, responding to page 4 required to inhibit the prepotent response to name the color in which the item was printed. The seconds needed for naming the colors of all 25 items per page (including potential error corrections) was measured and an interference score (time p.4 – ((time p.1 × time p.3)/(time p.1 + time p.3)) computed (Archibald and Kerns, 1999). Higher values indicate lower ability to inhibit the prepotent response.

#### Working Memory

In the Digit-Span Backwards Task (Petermann and Petermann, 2007), children were to repeat a heard sequence of digits in reverse order. Hence, the task requires children not only to recall the correct digits, but also to operate on this information when putting the digits in reverse order, which captures the central executive component of working memory (St Clair-Thompson and Allen, 2013; Giofrè et al., 2016; Donolato et al., 2017). Each trial consisted of 2 sequences of equal length (2 digits in the first sequence). If participants repeated at least 1 out of the 2 sequences within a trial correctly, the length of the sequences in the next trial was increased by 1 digit. After 2 wrong answers in a trial, the test was stopped. We computed the total number of sequences that had been repeated correctly.

#### Theory of Mind

In a computerized ToM cartoon task (Sebastian et al., 2012; originally developed by Völlm et al., 2006), children saw 12 cartoon stories (6 cognitive ToM, 6 affective ToM) that had been found to be of medium difficulty in a pilot study with 20 6–11 years-old children, who did not participate in the current study. Each story consisted of 5 black-and-white drawings showing two characters. The first 3 pictures appeared consecutively on a computer screen, followed by 2 drawings simultaneously, each of which depicted a possible ending to the story (inter-stimulus intervals: 1,000–3,000 ms; see Sebastian et al., 2012 for an example). We randomized the order of the stories and the side with the correct ending. We asked participants to select the “correct” ending that pointed to adequate ToM abilities by pressing the X-key (left-hand drawing) or the M-key (right-hand drawing; QWERTY keyboard). For a correct response, children had to infer the mental state of one protagonist and the appropriate response by the other. We chose the measure because it is economic and does not require high verbal abilities. In the present study, we included only cognitive ToM stories, in which the participant had to infer a protagonist’s intentions, desires, or beliefs. That way, it is comparable to previous studies on the relation between ToM and CP, which assessed similar aspects of ToM (Capage and Watson, 2001; Razza and Blair, 2009). We calculated the number of correct responses.

#### Conduct-Problem Symptoms

Parents rated their child’s CP symptoms on the 5 items (“Often lies or cheats”) of the accordant subscale of the Strength and Difficulties Questionnaire (SDQ; designed for 4–17-year olds; Goodman, 1997). Response options ranged from 1 *not true* to 3 *certainly true*. We calculated mean values.

#### Fluid Intelligence

In the Number-Symbol Test of the Wechsler Intelligence Scale for Children (Petermann and Petermann, 2007), participants were told to as quickly as possible redraw symbols (e.g., a half-moon) that were paired with either 5 simple figures (e.g., a cross with a circle inside; 6–7-year olds) or 9 digits (8–16-year olds). We calculated the number of correct symbols allocated within 120 s; 6–7 years olds gained extra points if they completed the task within 120 s. We computed standardized *T*-values.

#### ADHD Symptoms

Parents rated their child’s ADHD symptoms on the 5-item *Hyperactivity* subscale (e.g., “Is easily distracted”) of the SDQ (Goodman, 1997). Response options ranged from 1 *not true* to 3 *certainly true*. We calculated mean scores.

### Procedure

We assessed EF skills, ToM abilities, fluid intelligence, and ADHD symptoms at T1 and CP symptoms at T1 and T2. Measures were part of a task battery examining intrapersonal risk factors in childhood. Participants took part in two 50 min sessions which were conducted within a week of each other, respectively. Each child was assessed by a research assistant in a quiet room at school or at home. The order of the task battery was counterbalanced across participants. No effect of task sequence was found. Parents completed the questionnaires on paper or online. All measures and procedures were approved by the local Research Ethics Committee and the Ministry of Education. For each child, informed written consent was obtained from the parent/primary caregiver. As a reward, children received a cinema voucher.

### Statistical Analyses

Research questions were addressed by structural equation modeling (M*plus* 7.11; Muthén and Muthén, 2012). Missing data for the child measures (EF, ToM, fluid intelligence) were low (T1: ≤ 2.1%). Missing data for parent ratings (ADHD symptoms, CP symptoms) were ≤ 21.7% at T1 and ≤ 29.4% at T2. Missing values were accounted for by full-information maximum-likelihood (FIML) estimation. To ensure that this procedure did not result in biased estimations given the high percentage of missing data in the parent data, all analyses were compared to analyses in which data on at least one child variable were present. Because results of the FIML and the latter approach did not differ, we adopted the FIML procedure in order to include all participants into our analyses. Given that the χ^2^-statistic is sensitive to large samples, we evaluated the model fit according to the following cut-off criteria: comparative fit index (CFI) ≥ 0.95, root mean squared error of approximation (RMSEA) ≤ 0.08, standardized root mean residual (SRMR) ≤ 0.05 (for the model not including ToM), or weighted root-mean-square residual (WRMR) ≤ 1.0 (for the model including ToM; Geiser, 2010).

In all analyses, T1 EF skills, T1 ToM abilities, T1 CP symptoms, and T2 CP symptoms were entered as latent variables in order to adjust for random measurement error. All 3 indicators (flexibility, inhibition, cognitive aspects of working memory) loaded on one T1 EF factor with medium to high standardized factor loadings (range: 0.5–0.7, all *p*s ≤ 0.001). The standardized factor loadings of 4 of the 6 ToM items were medium to high (range: 0.5–0.6, *p*s ≤ 0.001), those of 2 items fell below a general cut-off value (<0.4) for the inclusion into a factor (Stevens, 2001). Therefore, the latent ToM factor consisted of 4 items. Because the ToM items were categorical and showed ceiling effects, a mean- and variance-adjusted weighted least square estimator (WLSMV) was used in the analysis including ToM. This estimator is suited for categorical data (Yu, 2002) and robust to violations of the assumption of normality (Muthén and Kaplan, 1985). Otherwise, we used the MLM estimator. The standardized factor loadings of 1 of the 5 CP-symptoms items fell below the general cutoff value (an item reflecting rather impersonal adverse conduct as compared to the other items reflecting rather interpersonally adverse behavior), those of the other 4 were medium to high (0.5–0.6, all *p*s ≤ 0.001 at T1 and T2). Thus, the latent CP symptoms factor consisted of the same 4 items at T1 and T2. An initial Confirmatory Factor Analysis including T1 EF, T1 ToM, T1 CP symptoms, and T2 CP symptoms with latent factors allowed to correlate confirmed the intended factor structure [χ^2^(90) = 122.694, *p* < 0.013, CFI = 0.985, RMSEA = 0.015, WRMR = 0.866].

Because age, gender, ADHD symptoms, and fluid intelligence are known to be associated with EF and/or CP symptoms (Lynam et al., 1993; Lahey and Waldman, 2007), these measures (assessed at T1) were included as manifest covariates. Furthermore, T1 CP symptoms were controlled for, assuming strict measurement invariance. Correlations between T1 predictors were allowed and estimated. Given the broad age range in our sample, we repeated the analysis without controlling for T1 age. Results, however, hardly differed for models including or excluding age as a covariate, indicating that the results apply to the total range of middle childhood. In the following, we report results for models including T1 age as a covariate. In this case, age did not add to the prediction of T2 CP symptoms.

To evaluate the mediation model, we examined whether the total effect and the indirect effect were significant, and whether the direct effect was significantly smaller than the total effect (partial mediation) or non-significant (full mediation; Geiser, 2010). To account for potential violations of assumptions of normal distributions, we used 10,000 bootstrap samples to estimate bias-corrected 95%-confidence intervals in order to evaluate the significance of the indirect effect.

## Results

### Descriptives

**FIGURE 1:**
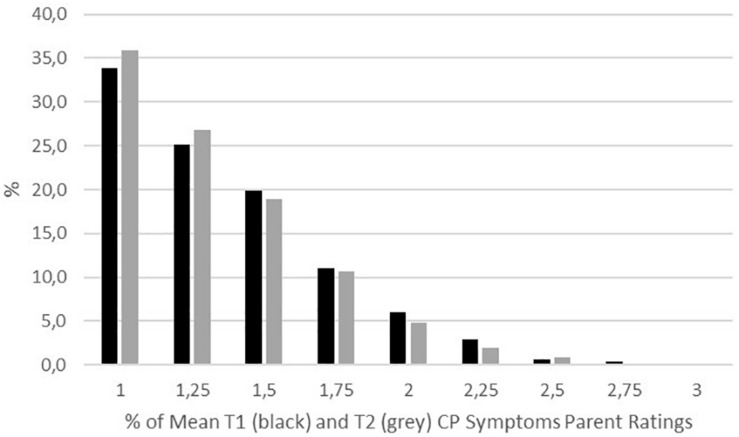
Percentage of mean parent ratings of T1 (black) and T2 (gray) CP symptoms (range: 1–3).

Table 1 shows descriptive statistics of all measures. CP symptoms at both measurement points and T1 ADHD symptoms yielded only low scores, indicating floor effects (see Figure 1). On average, there was no significant change in CP symptoms over approximately 1 year. The T1 ToM scores were rather high, indicating ceiling effects. For the other measures, medium to high scores were achieved. Given floor and ceiling effects for T1 ToM abilities, T1 CP symptoms, and T2 CP symptoms, we calculated ordinal alpha as the internal consistency measure for these variables.

**TABLE 1:** Descriptive statistics of CP symptoms, ToM, EF measures, and control variables.

	Range	α^1^	T1		T2		*t*
			*M*	*SD*	*M*	*SD*	
CP symptoms	1–3	0.75/0.73	1.36	0.37	1.33	0.34	1.2
ToM^a^	0–4	0.62	3.57	0.72			
Flexibility	0–22		15.57	4.68			
Inhibition^b^	0–89^c^		24.95	8.78			
Working memory	0–16		6.27	1.42			
ADHD symptoms	1–3	0.80	1.64	0.47			
Fluid intelligence^d^	27–80^c^		51.38	9.26			
Age	6–11		8.38	0.95			

*CP, conduct problems; ToM, cognitive Theory of Mind. ^1^Ordinal alpha for T1 and T2 CP symptoms and T1 ToM, Cronbach’s alpha for ADHD symptoms. ^a^Average number of correct trials. ^b^Interference measure (negatively polarized). ^c^Min and/or Max values are theoretically infinite, thus, table values are sample-specific. ^d^T-Value Number-Symbol-Test.*

### Correlations

Table 2 shows the zero-order correlations of the variables. T1 CP symptoms and T2 CP symptoms showed significant negative correlations with all T1 EF measures, and significant positive correlations with T1 ADHD symptoms. There were negative correlations of T1 CP symptoms with fluid intelligence, and of T2 CP symptoms with T1 ToM. T1 ToM abilities showed small but significant positive correlations with fluid intelligence and the EF measures inhibition and flexibility, but were unrelated to executive aspects of working memory. T1 age was significantly related to all measures, except for T1 CP symptoms, T2 CP symptoms, and T1 ADHD symptoms.

**TABLE 2:** Zero-order correlations of CP symptoms, ToM, EF measures, and control variables (upper figures) and partial correlations controlled for age (lower figures).

		1	2	3	4	5	6	7	8	9
1	CP symptoms T1		0.609***	−0.015***	−0.127***	0.095***	−0.098***	0.521***	−0.090***	0.010***
2	CP symptoms T2	0.597***		−0.074***	−0.146***	0.109***	−0.074***	0.374***	−0.040***	0.012***
3	ToM	–0.038	−0.068*		0.112***	−0.088***	0.069***	−0.005***	0.052***	0.096***
4	Flexibility	−0.134***	−0.151***	0.055		−0.328***	0.357***	−0.180***	0.157***	0.276***
5	Inhibition^a^	0.150***	0.118***	–0.045	−0.247***		−0.272***	0.162***	−0.263***	−0.347***
6	Working memory	−0.103***	−0.068*	0.008	0.293***	−0.188***		−0.121***	0.131***	0.239***
7	ADHD symptoms	0.505***	0.367***	–0.024	−0.202***	0.202***	−0.121***		−0.166***	0.031***
8	Fluid intelligence^b^	−0.115***	–0.041	00.49	0.183***	−0.310***	0.094**	−0.182***		−0.069***
9	Age									

*p = 0.05, **p = 0.01, ***p = 0.001; CP, conduct problems; ToM, cognitive Theory of Mind. ^a^Interference measure (negatively polarized). ^b^T-Value Number-Symbol-Test.

### Prediction of CP Symptoms From EF

To examine whether EF skills influence later CP symptoms while controlling for ADHD symptoms, we regressed T2 CP symptoms on T1 EF skills, controlling for T1 CP symptoms, age, gender, fluid intelligence, and ADHD symptoms [χ^2^(79) = 193.640, *p* < 0.001, CFI = 0.965, RMSEA = 0.030, SRMR = 0.031]. As expected, results showed a significant negative relation between T1 EF and T2 CP symptoms (β = −0.136, *p* = 0.043) despite high autocorrelations of T1 CP symptoms and T2 CP symptoms (β = 0.811, *p* ≤ 0.001) and after all covariates including ADHD symptoms had been taken into account.

### ToM Abilities as a Mediator of the Link of EF Skills and CP Symptoms

**FIGURE 2:**
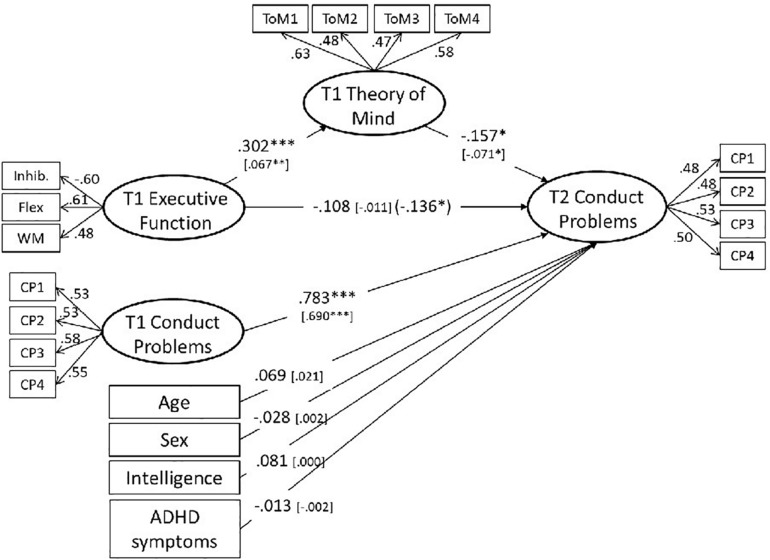
Structural equation model for the prediction of T2 CP symptoms from T1 EF mediated by T1 ToM and controlled for age, gender, fluid intelligence, and ADHD Symptoms. Digits in bracket: Effect of T1 EF on T2 CP symptoms without controlling for the mediating effect of T1 ToM. Correlation between T1 predictors allowed and estimated, but not displayed in the figure. Non-standardized path coefficients in square braquets. χ^2^(135) = 229.814, *p* < 0.001, CFI = 0.970, RMSEA = 0.021, WRMR = 0.963, *R*^2^ CP = 0.666. ^∗^*p* = 0.05, ^∗∗^*p* = 0.01, ^***^p = 0.001.

We then examined whether T1 ToM abilities mediate the relation of T1 EF skills and T2 CP symptoms, when controlling for T1 CP symptoms, age, gender, fluid intelligence, and ADHD symptoms. The model explained 66.6% variance in T2 CP symptoms [χ^2^(135) = 229.814, *p* < 0.001, CFI = 0.970, RMSEA = 0.021, WRMR = 0.963]. As expected, results showed a significant negative relation between T1 ToM abilities and T2 CP symptoms (β = −0.157, *p* = 0.023), despite high autocorrelations of T1 CP symptoms and T2 CP symptoms (β = 0.783, *p* < 0.001). Regarding the mediation, the model yielded a significant total effect (β = −0.155 [−0.284; −0.025], *p* = 027; 95% confidence intervals for exact effect sizes estimated via the bootstrapping procedure), a significant indirect effect from T1 EF skills via T1 ToM abilities on T2 CP symptoms (β = −0.047 [−0.091; −0.003], *p* = 0.049), and a non-significant direct effect from T1 EF skills on T2 CP symptoms (β = −0.108 [−0.250; 0.035], *p* = 0.162). Hence, findings indicate that the effect of T1 EF skills on T2 CP symptoms was fully mediated by T1 ToM abilities (Figure 2). We also examined the opposite way of effect with T1 EF mediating the links between T1 ToM and T2 CP symptoms. Given the cross-sectional relation between T1 EF and T1 ToM, the model showed a similar fit [χ^2^(135) = 232.443, *p* < 0.001, CFI = 0.970, RMSEA = 0.021, WRMR = 0.966] and explained 66.8% variance in T2 CP symptoms. However, despite a significant total effect (β = −0.161^∗^), neither the direct path from T1 ToM on T2 CP symptoms (β = −0.133, *p* = 0.052) nor the indirect path via EF (β = −0.028, *p* = 0.072) was significant. In addition, the path coefficient from T1 ToM to T1 EF was smaller than the path coefficient for the opposite link (β = 0.175^∗∗∗^ vs. β = 0.302^∗∗∗^). Based on these findings, the theoretically suggested model with ToM mediating the links between EF and CP seems somewhat more plausible than a model with EF mediating the link between ToM and CP.

## Discussion

The current study examined the longitudinal relations between EF skills, ToM abilities, and CP symptoms, as well as the potential mediating effect of ToM abilities on the link between EF skills and CP symptoms with longitudinal data from a large community sample in middle childhood. In line with our assumptions, higher EF skills and ToM abilities predicted less CP symptoms: The more a child was able to flexibly switch attention, to adapt the working memory, to inhibit impulses, and to infer others’ thoughts or intentions in computerized and playful paper-pencil tasks, the less antisocial behavior parents tended to report about 1 year later. Furthermore, in line with our predictions, the negative long-term relation between EF skills and CP symptoms was fully mediated by ToM abilities: Even though initial links of ToM with the other variables were small, in the structural equation models void of measurement error, the indirect negative effect from T1 EF skills via T1 ToM abilities on T2 CP symptoms was small, but significant. The initially significant direct negative effect from T1 EF skills on T2 CP symptoms was not significant anymore once ToM abilities were considered. The findings held constant despite controlling for potential inferring variables, including fluid intelligence or ADHD symptoms (Pennington and Ozonoff, 1996; Oosterlaan et al., 2005; Brocki et al., 2007). Hence, our empirical examination of the mediation model deepens the understanding of childhood CP symptoms by shedding light on the underlying developmental mechanisms.

### The Predictive Relation of EF Skills With Later CP Symptoms

The negative relation between EF skills and CP symptoms even when controlling for ADHD symptoms as found in child clinical samples (Toupin et al., 2000) can be transferred to a non-clinical sample with low levels of CP symptoms and ADHD symptoms as well as average EF skills. Our findings support the idea that various EF facets, such as the ability to inhibit impulsive behavior, to flexibly shift attention, and to keep track of all the key elements of a social situation all contribute to the prediction of CP symptoms (Moffitt, 1993, 2006; Ogilvie et al., 2011). Hence, the use of differential measures of EF skills in unselected samples in middle childhood may add to our understanding of the development of CP symptoms before adolescence. This relation could even be ascertained with EF scores obtained from tasks carried out by the children, whereas CP symptoms ratings were obtained from parent reports, indicating no artificial increases in effect sizes, because the multi-rater data assessment may account for biasing method effects.

### The Predictive Relation of ToM Abilities With Later CP Symptoms

Predictions of later CP symptoms by better abilities to infer others’ intentions, desires, or beliefs (cognitive ToM) even when controlling for other relevant variables support the idea that children with poor ToM abilities tend to falsely attribute negative intent to others and aggressively respond to these misinterpretations (Crick and Dodge, 1994). Due to inaccurate attributions of others’ mental states, these children may also underestimate the negative effects of maladaptive behaviors, such as lying or deceiving and hurting others (Happé and Frith, 1996). Accordingly, our findings complement a large body of research that found positive links between ToM abilities and social competence (Lalonde and Chandler, 1995; Cassidy et al., 2003; Razza and Blair, 2009; Caputi et al., 2012) as well as negative links with aggressive behavior (Capage and Watson, 2001) or antisocial behavioral problems (Hughes and Ensor, 2006) in non-clinical samples.

It has to be noted though, that other studies found no relations of (cognitive) ToM with conduct disorder in child clinical samples (Happé and Frith, 1996; Sutton et al., 2000), psychopathic traits in adults (Shamay-Tsoory et al., 2010), or proactive and reactive aggression in non-clinical child samples (Gomez-Garibello and Talwar, 2015; Austin et al., 2017). Some even found positive associations with bullying in adolescence (Caravita et al., 2010) or indirect aggression in preschool-age children with average or low levels of prosocial behavior (Renouf et al., 2010a). Hence, cognitive ToM abilities may enable an individual to manipulate or deceive others (Happé and Frith, 1996), particularly if affective ToM (i.e., the understanding of others’ feelings) is low (Shamay-Tsoory et al., 2010).

One reason for the contradicting findings on the links between ToM abilities and antisocial behavior may be that studies have focused on different aspects of ToM and social problems. For example, Renouf et al. (2010a, b) stressed the importance of the underlying function of the problematic behavior: Children’s ToM abilities may be negatively related to reactive aggression as an automatic and uncontrolled response toward alleged provocation, but positively related to proactive aggression as a means to reach one’s goals by, among others, deceiving or manipulating others. CP symptoms as defined in the current study encompassed both functions (e.g., “Often has bursts of anger; is quick-tempered” for reactive aggression, “Often lies or cheats” for proactive aggression) as well as other CP symptoms. Hence, in the present study it was not possible to sufficiently distinguish the two functions of aggression, that also tend to be positively correlated, further restricting possibilities to differentiate their distinct association with other variables (Card and Little, 2006). Furthermore, externalizing problem behavior, more closely related to CP symptoms, was negatively related, not related, or positively related to cognitive ToM, but consistently negatively related to affective ToM (Happé and Frith, 1996; Caravita et al., 2010; Shamay-Tsoory et al., 2010; Austin et al., 2017). Hence, future research should pay closer attention to the underlying subcomponents of both variables.

### The Mediation Model

So far, the assumption that EF deficits may promote lower ToM abilities and that lower ToM in turn promotes manifesting CP symptoms (Riggs et al., 2006) was only studied in preschool-age children with social competence as the outcome measure and with only partial support (Razza and Blair, 2009). The present study confirmed these findings in middle childhood and with CP symptoms as the outcome measure. These are important findings; given that middle childhood requires high levels of EF and ToM (Lalonde and Chandler, 1995), low levels of CP symptoms can be assumed to be frequent (Ravens-Sieberer et al., 2008) even among children from a community sample. In addition, ToM abilities fully mediated the link between EF skills and CP symptoms beyond other important control variables and CP symptoms stability. ToM added to the explanation of 66.6% variance in T2 CP symptoms beyond the high stability of CP symptoms. This supports the assumption that EF impairments are related to difficulties in attributing others’ mental states which, in turn, lead to higher levels of CP symptoms (Riggs et al., 2006).

Our findings diverge from those of a previous study in a clinical sample of elementary-school age children with attention and behavior problem that did not find a relation between ToM abilities and social problems once EF skills were controlled for (Fahie and Symons, 2003). It has been suggested that individuals with extreme behavior problems possess intact (instead of low) ToM capacities, which allow them to deceive or manipulate others (Sutton et al., 1999), and if so, the present mediation model may not be valid in such extreme groups. Furthermore, when children are clinically referred for behavior *and* attention problems, this may confound maladaptive behaviors resulting from deficits in EF skills with those attributed only to ADHD. Further research in clinical and non-clinical samples of different ages is needed to further disentangle the specific contributions of the potential risk factors in different groups.

### Limitations and Implications

The strengths of the present study include the large population-based sample size, the use of multiple measures for EF, the consideration of fluid intelligence and ADHD symptoms as control variables, as well as the use of longitudinal data to examine the research question. Limitations include the reliance on two measurement points when testing a mediation model, limiting the interpretability of our findings (Wiedermann and von Eye, 2015). Because the link between EF skills and ToM abilities in middle childhood, however, is well-established in behavioral research (Austin et al., 2014; Devine and Hughes, 2014), we think that it was reasonable to assess these two variables simultaneously and to concentrate on testing both their longitudinal effects on CP symptoms about 1 year later. Second, we addressed the question whether early EF skills would predict later CP symptoms over and above the effects of ADHD symptoms, but did not include an ADHD diagnosis according to DSM criteria. However, because children in the present study came from a community sample, symptoms (CP, ADHD, impairments in EF skills and ToM abilities) can be expected to be less severe, and an ADHD diagnosis was not necessarily needed. Third, there was a ceiling effect and limited variance on the ToM cartoons, suggesting that the task was easy for children in the examined age range, whereas there was a floor effect on the CP symptoms measure, which was to be expected in an unselected sample. We used the WLSMV estimator that is robust against skewed distributions (Muthén and Kaplan, 1985) to account for these effects. Two of the ToM items did not load on the latent ToM factor, further reducing the number of items in this measure. Fourth, despite the relatively broad age range in our study and despite further development of both EF skills and ToM abilities throughout middle childhood, we were unable to provide a multi-group model examining age as a potential moderator. Finally, internal consistencies for both ToM abilities and CP symptoms were relatively low. This may be due to the ceiling or floor effects, the limited response options (Gadermann et al., 2012), and the shortage of the scales, respectively.

Thus, future research may want to apply more reliable measures for ToM abilities and CP symptoms that can be used in middle childhood. Given previous findings on differential relations of affective and cognitive ToM with a number of externalizing problem behaviors in childhood (Anastassiou-Hadjicharalambous and Warden, 2008; Austin et al., 2017), future research may further disentangle specific effects of subcomponents of ToM abilities (including affective and cognitive ToM). Similarly, given recent research pointing to the diversity of EF (Karr et al., 2018), future research may also want to further disentangle the potential specific effects of different EF skills in mediation models. Finally, research could also consider other outcome measures, such as (proactive and reactive) aggression, or other mediating variables, such as empathy. Future research testing a similar mediation model may want to employ three points of measurement that will at the same time allow testing for the developmental sequence of EF skills and ToM abilities. It may also examine the potential moderating role of age in this particular age range. Finally, future research could show whether fostering EF skills and ToM abilities in children with and without ADHD symptoms may buffer the positive association of ADHD and CP symptoms.

## Conclusion

In conclusion, the current study supports the idea that lower EF skills play a role in the occurrence of CP symptoms about 1 year later in non-selected samples from middle childhood. Our results also indicate that although EF skills may influence CP symptoms, their influence is indirect through cognitive ToM abilities and tends to be small. However, given the small variance in many variables in our community sample as well as the high stability of CP symptoms, this finding still seems noteworthy. Thus, the complex developmental relations between social-cognitive competencies and CP symptoms in childhood require the consideration of mediating and moderating effects (Riggs et al., 2006). In line with recent research pointing to at least partial independence of EF skills and ToM abilities (Wade et al., 2018), the present findings indicate that for the prevention of CP symptoms, it is not sufficient to promote either EF skills or ToM abilities. Both should be targeted when trying to prevent future norm violations in children or when aiming at fostering prosocial behavior.

## Data Availability Statement

The datasets for this manuscript are not publicly available because the data are part of a larger longitudinal study with ongoing publications. Stricter guidelines for data protection also interfere with the publication of larger parts of the data set. Requests to access the datasets should be directed to the corresponding author.

## Ethics Statement

The studies involving human participants were reviewed and approved by Ethics committee of the University of Potsdam. Written informed consent to participate in this study was provided by the participants’ legal guardian/next of kin.

## Author Contributions

All authors listed have made a substantial, direct and intellectual contribution to the work, and approved it for publication.

## Conflict of Interest

The authors declare that the research was conducted in the absence of any commercial or financial relationships that could be construed as a potential conflict of interest.
